# The expression pattern of pyroptosis-related genes predicts the prognosis and drug response of melanoma

**DOI:** 10.1038/s41598-022-24879-y

**Published:** 2022-12-13

**Authors:** Bin Zhou, Shanshan Sha, Juan Tao, Jun Li, Chen Shen, Jinjin Zhu, Lulu Tan, Liyun Dong, Changzheng Huang

**Affiliations:** 1 Hubei Engineering Research Center of Skin Disease Theranostics and Health, Department of Dermatology, Union Hospital, Tongji Medical College, Huazhong University of Science and Technology (HUST), Wuhan, 430022 China; 2 Department of Dermatology, The Central Hospital of Wuhan, Tongji Medical College, Huazhong University of Science and Technology (HUST), Wuhan, 430014 China; 3 Research Center for Tissue Engineering and Regenerative Medicine, Union Hospital, Tongji Medical College, Huazhong University of Science and Technology, Wuhan, 430022 China

**Keywords:** Skin cancer, Tumour biomarkers, Tumour immunology

## Abstract

Cutaneous melanoma (CM, hereafter referred to as melanoma) is a highly malignant tumor that typically undergoes early metastasis. Pyroptosis, as a special programmed cell death process that releases inflammatory factors and has been widely studied in tumors, but its role in melanoma has not been fully elucidated. In this study, we examined the relationship between pyroptosis and the prognosis of melanoma through bioinformatic analysis of RNA-sequencing data. Our results demonstrated that pyroptosis is a protective factor associated with melanoma prognosis. A higher pyroptosis score was associated with a more favorable overall survival. We used weighted gene co-expression networks analysis (WGCNA) to establish an effective prognosis model based on 12 pyroptosis-related genes. We then validated it in two independent cohorts. Furthermore, a nomogram combining clinicopathological characteristics and a pyroptosis-related gene signature (PGS) score was designed to effectively evaluate the prognosis of melanoma. Additionally, we analyzed the potential roles of pyroptosis in the tumor immune microenvironment and drug response. Interestingly, we found that the elevated infiltration of multiple immune cells, such as CD4^+^ T cells, CD8^+^ T cells, dendritic cells, and M1 macrophages, may be associated with the occurrence of pyroptosis. Pyroptosis was also related to a better response of melanoma to interferon-α, paclitaxel, cisplatin and imatinib. Through Spearman correlation analysis of the 12 pyroptosis-related genes and 135 chemotherapeutic agents in the Genomics of Drug Sensitivity in Cancer database, we identified solute carrier family 31 member 2 (*SLC31A2*) and collagen type 4 alpha 5 chain (*COL4A5*) as being associated with resistance to most of these drugs. In conclusion, this PGS is an effective and novelty prognostic indicator in melanoma, and also has an association with the melanoma immune microenvironment and melanoma treatment decision-making.

## Introduction

Melanoma is the deadliest known skin tumor, and its incidence has increased dramatically across the world over the past few decades^1–3^. Melanoma originates from melanocytes or melanoblasts, and the melanogenesis can affect the sensitivity of melanoma to radiotherapy and chemotherapy^4–8^. Early melanomas can be surgically excised to obtain a good prognosis, but these cancers are very prone to metastasis and recurrence, thus causing a low survival rate and poor prognosis^9,10^. Although the systemic treatments provided by targeted therapies and immunotherapies have been widely used in patients with metastatic melanoma^11^, nearly 50% of these cases either show no response or eventually develop resistance^11–13^. Currently, the classification of melanoma based on pathologic characteristics cannot accurately evaluate the prognosis and there is a dire lack of biomarkers for evaluating the drug response^14^. The identification of novel and efficient prognostic and predictive factors is therefore urgently needed to help improve the clinical management of melanoma.

The dysregulation of cell death processes is a hallmark of cancer and is related to the resistance of tumor cells to cancer therapies^15,16^. Programmed cell death (PCD) is orchestrated by precise molecular pathways that maintain tissue homeostasis through the clearing of unwanted cells^17^. Many previous studies have found that cancer cells can downregulate PCD signaling to evade cell death, thus promoting their invasiveness and development of drug resistance^18–20^. PCD induction is a potential cancer therapeutic strategy and may improve the efficacy of immunotherapy^21^. Pyroptosis is a newly discovered subtype of PCD, and was first observed in *Shigella flexneri*-infected macrophages^22^. Differing from apoptosis, pyroptosis is caspase-dependent and is accompanied by gasdermin (GSDM) cleavage and the release of proinflammatory factors such as Interleukin-1β (IL-1β) and IL-18^23^. Pyroptosis can therefore protect the host from pathogen infection but also induce pathological diseases^24^, including atherosclerosis^25^, neuroinflammation^26^ and autoimmune disorders^27^. However, the role of pyroptosis in tumor progression remains controversial. Although inducing pyroptosis may kill cancer cells, the inflammatory environment that would be created by this can also promote tumorigenesis and metastasis^28^. Pan-cancer analysis has revealed that the pyroptosis executor gasdermin D (GSDMD) is a prognostic marker in melanoma^29^. However, the inflammasome eucine-rich repeat protein 1 (NLRP1) that can induce pyroptosis was also found to promote melanoma growth^30^. Previous studies have reported that pyroptosis-related genes play a dual role in melanoma^31^. Little is known to date however about the effect of pyroptosis on the progression and prognosis of melanoma, or its potential effect on the immune microenvironment and drug response of melanoma.

In this study, we have comprehensively explored the association between pyroptosis and the prognosis, immune microenvironment, and drug response of melanoma by analyzing transcriptome data obtained from the Gene Expression Omnibus (GEO) and The Cancer Genome Atlas (TCGA) datasets. Single sample gene set enrichment analysis (ssGSEA) was implemented to assess the impact of pyroptosis on the prognosis of melanoma. Using weighted coexpression network analysis (WGCNA), a novel pyroptosis-related gene signature (PGS) was constructed to evaluate the prognosis of melanoma. Moreover, we established a nomogram to further illustrate the prognostic value of PGS in melanoma. Finally, the xCell and Genomics of Drug Sensitivity in Cancer database (GDSC) were utilized to investigate the correlation between the PGS and the immune microenvironment and drug response in melanoma.

## Results

### Pyroptosis is a vital protective process associated with the prognosis of melanoma

**Figure 1:**
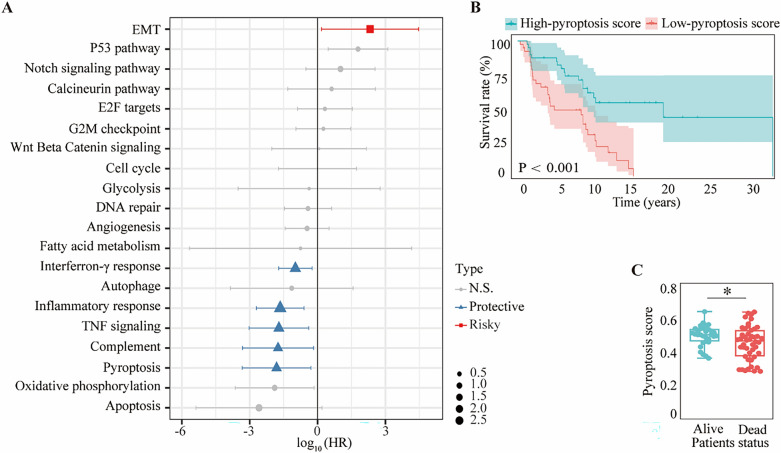
Pyroptosis is related to melanoma overall survival (OS). (**A**) Hazard ratio (HR) for each of 20 cancer hallmarks of prognosis on a forest plot. (**B**) Kaplan–Meier OS curves for melanoma patients with high and low pyroptosis scores. (**C**) Comparison of pyroptosis scores in living and dead melanoma patients. Statistical analysis: *p < 0.05. This figure was created using R software version 4.0.3^32^ (https://www.r-project.org/).

A flowchart of the current study design is provided in Supplementary Fig. S1. ssGSEA was used to evaluate the RNA-sequencing data of the 77 melanoma samples obtained from the GSE54467 dataset. The link between melanoma prognosis and cancer hallmarks were assessed by Cox regression analysis. Cancer hallmark-related gene sets were downloaded from the MSigDB database (Supplementary Table S1). These results revealed that pyroptosis (hazard ratio [HR] : 1.541 × 10 (− 2); 95% CI 5.221 × 10 (− 4) − 5.101 × 10 (− 1); *p* < 0.05), complement (HR: 1.792 × 10 (− 2); 95% CI 5.077 × 10 (− 4) − 6.667 × 10 (− 1); *p* < 0.05), and tumor necrosis factor (TNF) signaling (HR: 1.952 × 10 (− 2); 95% CI 9.136 × 10 (− 4) − 4.077 × 10 (− 1); *p* < 0.05) were strongly related to a favorable overall survival (OS) of the melanoma patients (Fig. 1A; Supplementary Table S2). Analysis of the expression data from the TCGA dataset confirmed that autophagy (HR: 3.177 × 10 (− 4); 95% CI 3.026 × 10 (− 6) − 3.117 × 10 (− 2); *p* < 0.001), apoptosis (HR:7.388 × 10 (− 4); 95% CI 2.479 × 10 (− 5) − 2.224 × 10 (− 2); *p* < 0.05), and pyroptosis (HR: 3.271 × 10 (− 3); 95% CI 4.463 × 10 (− 4) − 2.131 × 10 (− 2); *p* < 0.001) were also strongly associated with an improved OS (Supplementary Fig. S2A; Supplementary Table S3). Overall, pyroptosis was found to be the most significant protective factor associated with a favorable OS in melanoma by these analyses. Using the cutoff value determined by the median pyroptosis score, the melanoma patients were divided into high and low pyroptosis score groups. A high pyroptosis score was linked to a more favorable overall survival and longer OS time, the latter could be regarded as a hallmark of the former (Fig. 1B; Supplementary Fig. S2B). Meanwhile, the pyroptosis scores in the living melanoma patients were distinctly higher than those in the dead cases (Fig. 1C; Supplementary Fig. S2C). These results indicates that pyroptosis is a crucial protective factor linked with the prognosis of melanoma.

### Construction of a pyroptosis-related gene signature for melanoma

**Figure 2:**
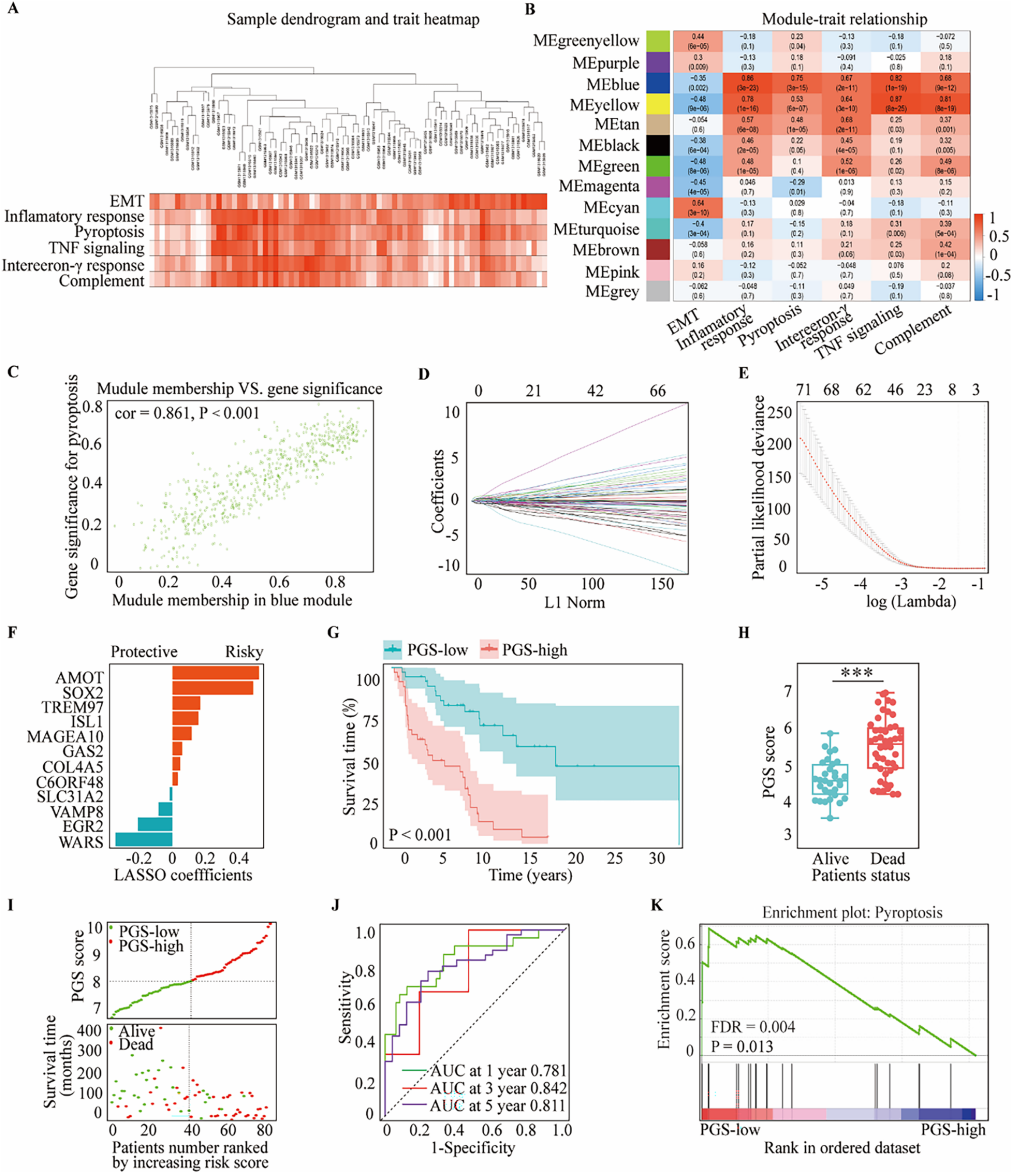
Construction of the PGS using the melanoma cases in the GSE54467 dataset. (**A**) Clustering dendrogram of the 77 samples using WGCNA. (**B**) Heatmap of the interrelation between cancer hallmarks and the modules. (**C**) Interrelation between the blue module and pyroptosis. (**D**) LASSO coefficient results for the relationship between pyroptosis-related differentially expressed genes and melanoma prognosis. (**E**) Penalty parameter λ selection via tenfold cross-validation in the LASSO model. (**F**) 12 pyroptosis-related genes determined from LASSO coefficients. (**G**) Kaplan–Meier OS curves for patients in the PGS-low and -high groups. (**H**) Comparison of PGS scores in living and dead patients. (**I**) Distribution of PGS scores, patient status and OS periods. (**J**) Time-dependent ROC curves at 1, 3 and 5 years. (**K**) GSEA of the pyroptosis pathway in the PGS-low and -high groups. Statistical analysis: ***p < 0.001. This figure was created using R software version 4.0.3 (https://www.r-project.org/) and GSEA software 4.2.1 (https://www.gsea-msigdb.org/gsea/index.jsp)^33^.

To next investigate whether pyroptosis could evaluate the prognosis of melanoma, WGCNA was used to compare the co-expression patterns between whole-transcriptome profiling data and pyroptosis scores (Fig. 2A). An optimal soft threshold of 7 was set to build a scale free network (Supplementary Fig. S3A,B) and 13 modules were thereby recognized (Supplementary Fig. S3C). The blue module closely related to pyroptosis was selected for further analysis (Fig. 2B,C). The most effective prognostic markers within the module were chosen using the least absolute shrinkage and selection operator (LASSO) Cox regression analysis. Twelve genes (*AMOT*, *SOX2*, *TREM97*, *ISL1*, *MAGEA10*, *GSA2*, *COL4A5*, *C6ORF48*, *SLC31A2*, *VAMP8*, *EGR2* and *WARS*) were identified and applied to the construction of an optimal pyroptosis-related gene signature (PGS) model (Fig. 2D,E). Among these genes, *AMOT*, *SOX2*, *TREM97*, *ISL1*, *MAGEA10*, *GSA2*, *COL4A5* and *C6ORF48* were linked with a worse melanoma prognosis, while the rest were found to be related to a better prognosis (Fig. 2F). The PGS score of every patient was calculated on the basis of the expression levels of the 12 aforementioned genes. Patients were stratified into two groups in accordance with the median cutoff value of the PGS scores, those above this median were the PGS-high group and vice versa. Principal component analysis enabled us to affirm the rationality of this division (Supplementary Fig. S4). Kaplan–Meier analysis revealed that the PGS-low group displayed a better OS (*p* < 0.001; Fig. 2G) and that the dead patients had higher PGS scores (*p* < 0.001; Fig. 2H). These findings were consistent with the survival analysis data (Fig. 2I). The areas under the receiver operating characteristic (ROC) curves for the 1-, 3-, and 5-year OS outcomes associated with the PGS scores were 0.781, 0.842, and 0.811, respectively (Fig. 2J). The result indicated that our PGS has a high accuracy in predicting 1-, 3-, and 5-year survival rate. Gene set enrichment analysis (GSEA) indicated that the genes expression of the PGS-low group was associated with pyroptosis induction (*p* < 0.001; Fig. 2K).

### The PGS score is an independent prognostic indicator in melanoma

**Figure 3:**
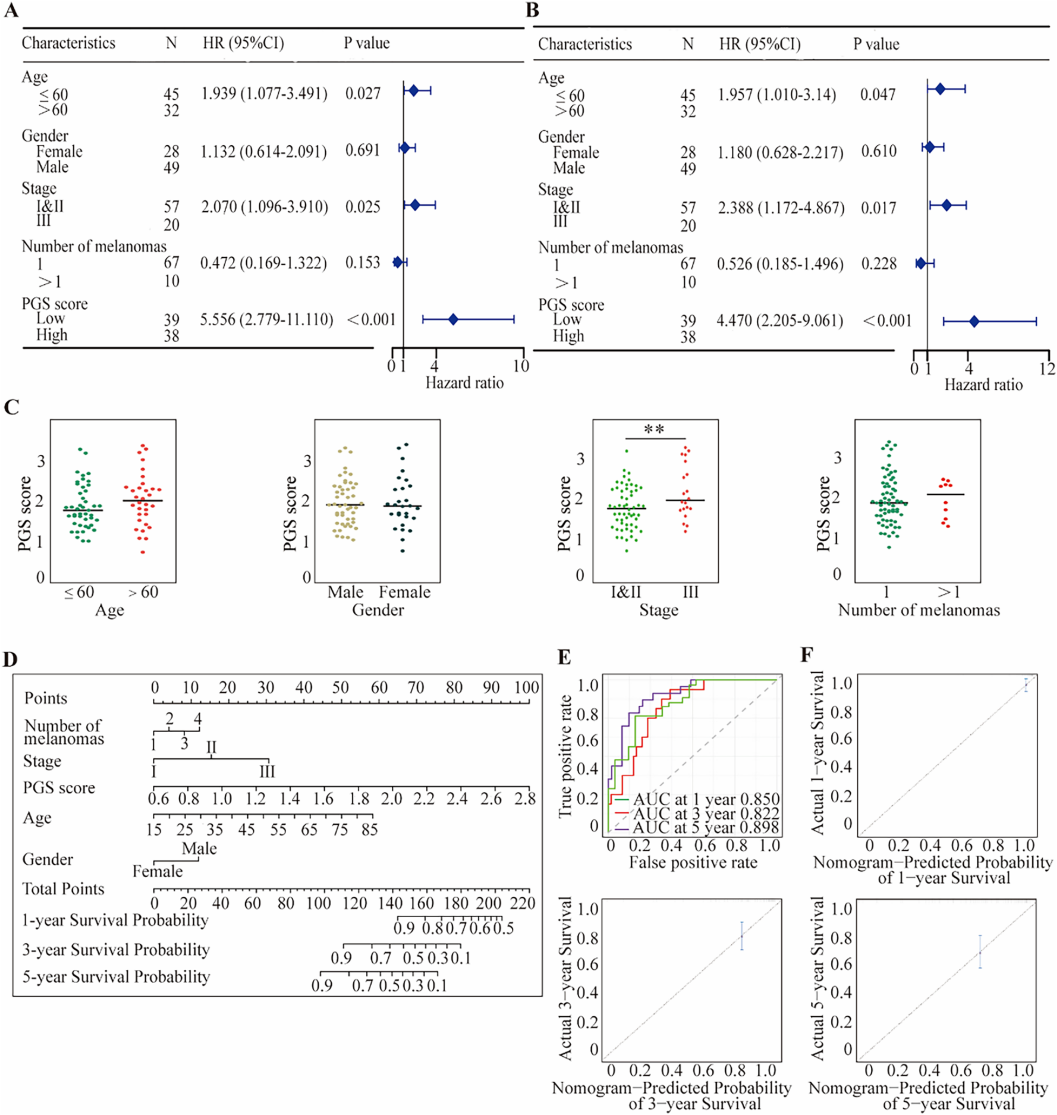
PGS scores are associated with the melanoma prognostic index in the GSE54467 samples. The PGS was found to be an independent risk factor for melanoma according to both univariate (**A**) and multivariate (**B**) Cox analysis. (**C**) Interrelationships between the PGS score and clinicopathological characteristics of the melanoma study patients. (**D**) Nomogram based on the PGS scores and clinicopathological characteristics. (**E**) Time-dependent ROC curves at 1, 3 and 5 years. (**F**) Calibration plot of the comparison between the nomogram-predicted and actual 1, 3 and 5-year OS periods. Statistical analysis: **p < 0.01. This figure was created using R software version 4.0.3 (https://www.r-project.org/).

The prognostic value of the PGS score was assessed in the training cohorts (GSE54467). Univariate and multivariate Cox regression analysis revealed that PGS score was an independent risk factor for melanoma (Fig. 3A,B; Supplementary Table S4) and that a high PGS score was associated with an advanced clinical stage of melanoma (Fig. 3C). Interestingly, the patients in this dataset were only labeled with three melanoma stage I, II, and III but no detailed TNM stage information. We further visualized the prognostic value of the PGS score in melanoma by generating a nomogram for predicting individual OS probabilities through a combination of clinicopathological characteristics (gender, age, tumor stage and number of primary melanoma) and the PGS scores of the patients (Fig. 3D). It was noteworthy that young and early-stage melanoma patients had better outcomes, but gender and number of melanomas played meaningless roles in survival (Supplementary Fig. S5A–D). The findings from these analyses indeed suggested that the PGS score is an important independent indicator of the prognosis of melanoma. The areas under the ROC curves for the 1-, 3-, and 5-year OS periods in the nomogram were 0.850, 0.822, and 0.898, respectively and PGS was more closely associated with prognosis than common clinical characters (Fig. 3E; Supplementary Fig. S5E). In comparison with other models, the nomogram exhibited the most significant ability for OS prediction in our melanoma sample population. The probabilities for 1-, 3-, and 5-year survival revealed substantial uniformity between the observed values and those predicted by the nomogram (Fig. 3F).

### Verification of the pyroptosis-related gene signature in melanoma

**Figure 4:**
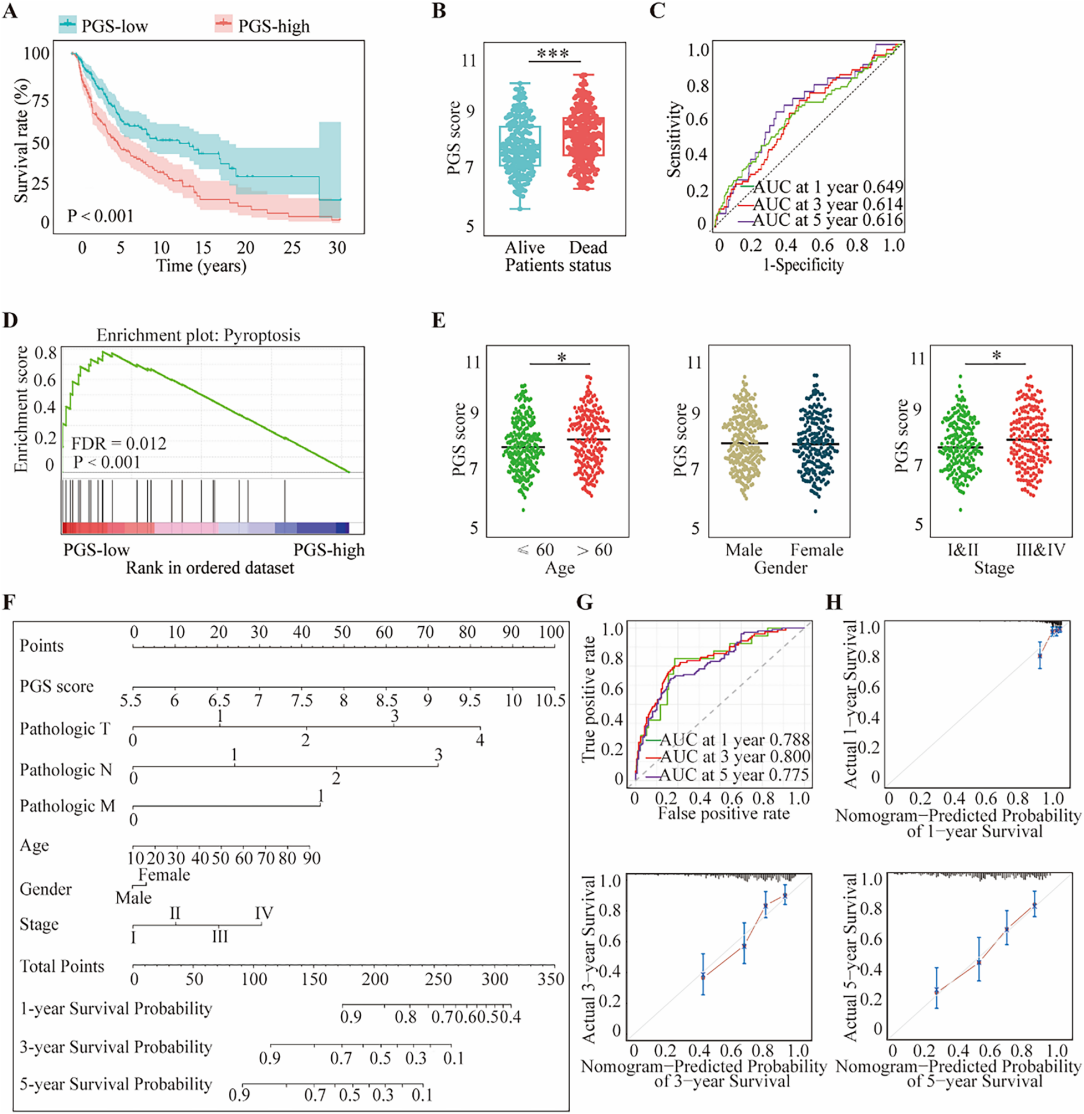
Verification of the PGS scoring system in the TCGA cohort. (**A**) Kaplan–Meier OS curves for patients in the PGS-low and -high groups. (**B**) Comparison of the PGS scores in the melanoma patients. (**C**) Time-dependent ROC curves at 1, 3 and 5 years. (**D**) GSEA evaluation of the pyroptosis pathway in the PGS-low and -high groups. (**E**) Correlation of PGS score with clinicopathological characteristics. (**F**) Nomogram constructed according to the PGS scores and clinicopathological characteristics. (**G**) Time-dependent ROC curves at 1, 3 and 5 years. (**H**) Calibration plot of the contrast between the actual 1, 3 and 5-year and nomogram-predicted OS outcomes. Statistical analysis: *p < 0.05, ***p < 0.001. This figure was created using R software version 4.0.3 (https://www.r-project.org/) and GSEA software 4.2.1 (https://www.gsea-msigdb.org/gsea/index.jsp).

To further verify the prognostic value of the PGS score, two independent melanoma cohorts (TCGA cohort and GSE19234) were utilized. The two datasets were independent of GSE54467 and included age, gender, and primary diagnosis information. Both PGS-low and -high groups contained patients at different stages or receiving different treatments, and were not artificially interfered with the groups through clinical characters. Among the TCGA cases, Kaplan–Meier analysis certified that the PGS-low group displayed a longer OS (*p* < 0.001; Fig. 4A) and that the dead patients had a higher PGS score (*p* < 0.001; Fig. 4B). The areas under the ROC curves for the 1-, 3-, and 5-year OS outcomes, stratified by the PGS score, were 0.649, 0.614, and 0.616, respectively (Fig. 4C). Similar results were obtained for the GSE19234 cases (Supplementary Fig. S6A,B) for which the areas under the ROC curve for the 1-, 3-, and 5-year OS in association with the PGS score were 0.707, 0.600, and 0.595, respectively (Supplementary Fig. S6C). The result validated the PGS could predict 1-, 3-, and 5-year survival rate precisely. The GSEA results indicated that pyroptosis induction was related to gene expression in the PGS-low group (*p* < 0.001; Fig. 4D). The clinical information of patients in the TCGA cohort included age, gender, detailed TNM stage information and four melanoma stageI, II, III, and IV. Further analyses demonstrated that a high PGS score had an association with more advanced melanoma stage. Interestingly, age was also found to be associated with the PGS score (Fig. 4E). Further study demonstrated that young and early-stage melanoma patients had longer survival time, but gender did not show a significant effect on survival (Supplementary Fig. S7A–F). A nomogram was established by combining the PGS score and the clinicopathological characteristics of the melanoma patients (age, gender, and tumor stage) in the TCGA dataset (Fig. 4F). The areas under the ROC curves for the 1-, 3-, and 5-year OS outcomes of the nomogram were 0.788, 0.800, and 0.775, respectively, demonstrated PGS score was more tightly related to prognosis (Fig. 4G; Supplementary Fig. S7G). The probabilities for the 1-, 3-, and 5-year OS values displayed reasonable agreement (Fig. 4H).

### Correlation between pyroptosis and the tumor microenvironment of the melanoma lesions

**Figure 5:**
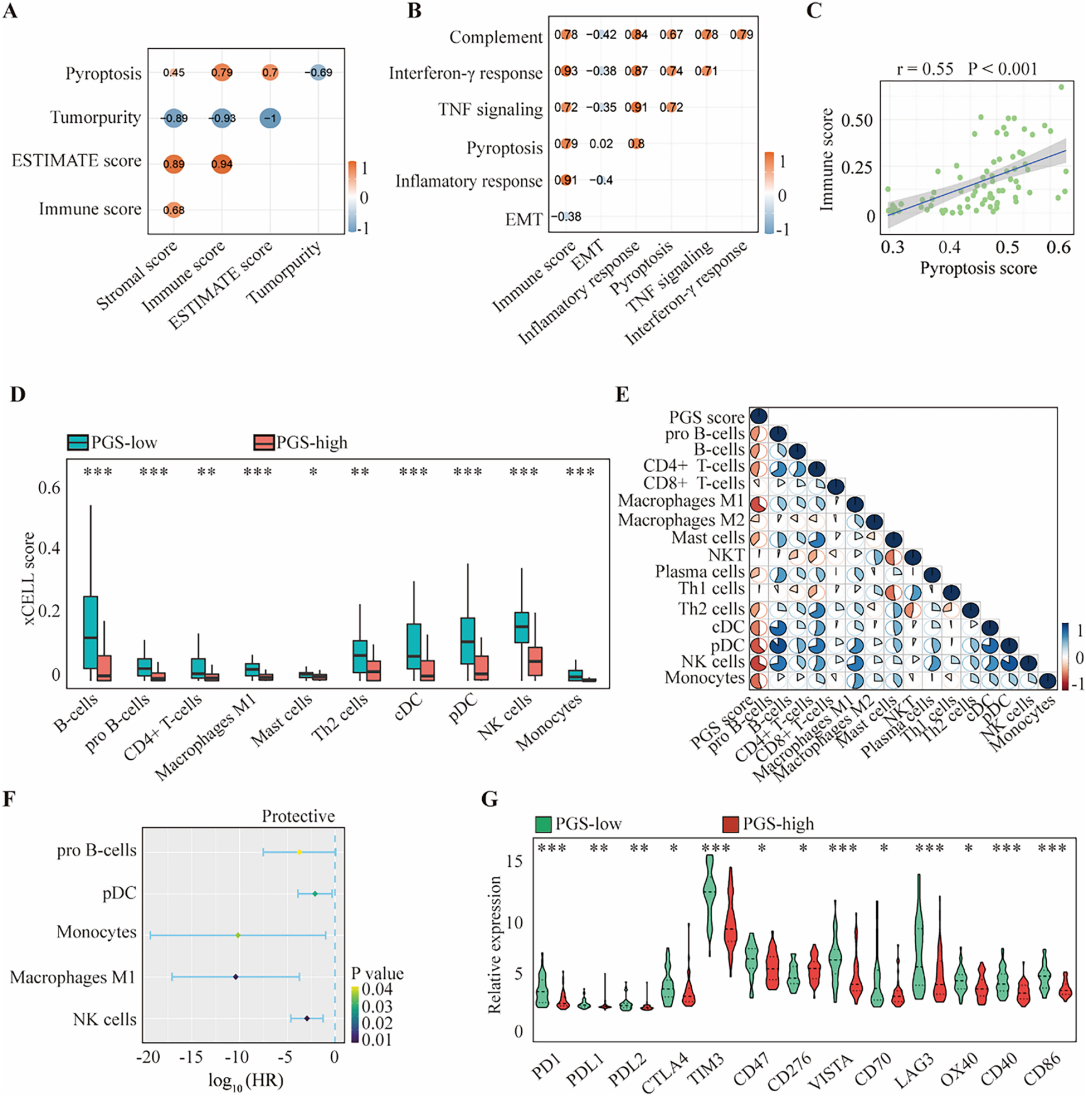
The interrelationship between the PGS and immune cells infiltration. (**A**) Spearman association analysis between immune cells, stromal cell infiltration and pyroptosis. (**B**) The interrelationship between cancer hallmarks and pyroptosis. (**C**) The interrelationship between immune score and pyroptosis score. (**D**) The infiltration level of 10 immune cells in PGS-low and -high groups. (**E**) The association between the PGS score and various immune cells. (**F**) Multivariate Cox regression analysis to identify various prognostic immune cell types in the forest plot of the HR values. (**G**) The expression pattern of immune checkpoint-related and immune active genes in the PGS-low and -high groups. Statistical analysis: *p < 0.05, **p < 0.01, ***p < 0.001. This figure was created using R software version 4.0.3 (https://www.r-project.org/).

To further illustrate the underlying role of pyroptosis in tumor microenvironment (TME), correlation analysis between pyroptosis and the immune and stromal cells profiles was performed. The results demonstrated that immune score was strongly with pyroptosis (Fig. 5A). Moreover, pyroptosis was found to be positively correlated with the immune score (Fig. 5B,C), indicating its possible mediation by immune cells in the tumor microenvironment. For TME infiltrating cell types, we used the xCELL to abtain enrichment scores for 35 immune-related and 13 stromal-related cell types. Those immune-related cells with different level between PGS-low and -high groups (p < 0.05) were selected. The results revealed that B cells, pro B cells, CD4^+^ T cells, M1 macrophages, mast cells, Th2 cells, cDCs, pDCs, NK cells, and monocytes, were found to be associated with the PGS score in melanoma and show a significantly higher prevalence in the PGS-low group (Fig. 5D). A correlation matrix between the PGS scores and these immune cells revealed that pro B cells, pDCs, monocytes, M1 macrophages and NK cells were not only more abundant in the PGS-low group (Fig. 5E), but also related to better OS (Fig. 5F). In addition, the level of CD8^+^ T cells in PGS-low group increased significantly using MCPcounter and Cibersort, suggesting a possible role of CD8 ^+^ T cells in the TME (Supplementary Fig. S8). To explore the immune landscape in the tumor microenvironment of the two groups, the expression levels of immune checkpoint-related genes were analyzed, including *PD1*, *PDL1*, *PDL2*, *CTLA4*, *TIM3*, *CD47*, *CD276*, *VISTA*, *CD70* and *LAG3.* Immune active genes such as *OX40*, *CD40* and *CD86* were also evaluated. The Wilcoxon test indicated that the PGS-low group had a higher expression of these genes (Fig. 5G). Our results further showed that there was no significant correlation between stromal cells and pyroptosis (Supplementary Fig. S9). These aforementioned findings may suggest that pyroptosis is related to the infiltration of various immune cells, and that it affects the immune status of the melanoma microenvironment. Moreover, our current data suggest that pyroptosis may affect the drug response of melanoma lesions by mediating their immune microenvironment.

### Correlation between the PGS score and drug response in melanoma

**Figure 6:**
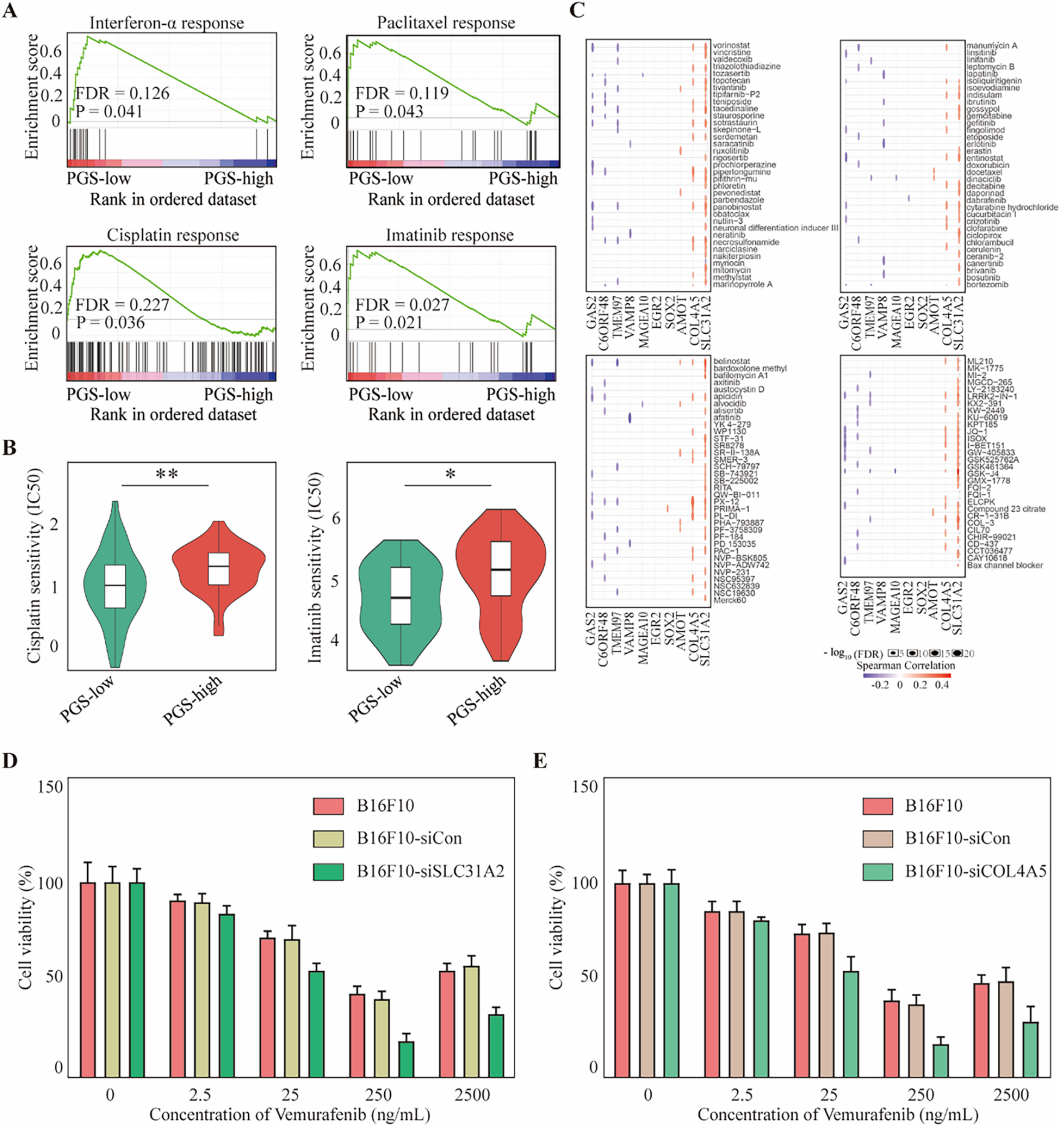
Correlation between the PGS score and chemotherapeutic drug response in melanoma. (**A**) GSEA assessment of interferon-α, paclitaxel, cisplatin and imatinib response pathways in the PGS-low and -high groups. (**B**) Sensitivity analysis of cisplatin and imatinib between the PGS-low and -high groups. (**C**) Spearman correlation analysis between the 10 pyroptosis genes and 135 chemotherapeutic drugs. (**D**,**E**) Cell viability of B16F10 melanoma cells transfected with siCon, siSLC31A2, and siCOL4A5 and naive cells treated with different concentrations of vemurafenib. Statistical analysis: *p < 0.05, **p < 0.01. This figure was created using GSEA software 4.2.1 (https://www.gsea-msigdb.org/gsea/index.jsp).

GSEA evaluations of gene sets associated with drug responses in melanoma demonstrated that the interferon-α response, paclitaxel response, cisplatin response and imatinib response gene sets were overexpressed in the PGS-low group (Fig. 6A). Drug response-related gene sets were downloaded from the The Molecular Signatures Database (MSigDB) (Supplementary Table S5). Additionally, patients in the PGS-high group exhibited a higher 50% inhibitory concentration (IC_50_) of cisplatin and imatinib (Fig. 6B). Spearman correlation analysis of the 12 pyroptosis-related genes and 135 chemotherapeutic drugs in the Genomics of Drug Sensitivity in Cancer database revealed that the expression of the solute carrier family 31 member 2 (*SLC31A2*) and collagen type 4 alpha 5 chain (*COL4A5*) was related to the resistance to most chemotherapies (Fig. 6C). The cell counting Kit-8 (CCK-8; Sigma-Aldrich, Shanghai, China) assay demonstrated that *SLC31A2* and *COL4A5* are tightly associated with vemurafenib-resistance (Fig. 6D,E; Supplementary Table S6). These outcomes further indicate that the PGS score is a useful tool for predicting the drug response in melanoma patients in a clinical setting.

## Discussion

Pyroptosis is a form of programmed cell death activated by inflammasomes and caspase^34^, and its potential role in tumor prognosis and therapeutic strategies for cancer is being explored by various research groups. The effect of pyroptosis in melanoma is obscure at present, however. In our present study, we have found that pyroptosis is a protective factor that is associated with a more favorable prognosis in melanoma, it significantly correlates with a higher survival rate and lower mortality in the affected patients. We then constructed a novel melanoma prognosis model based on 12 pyroptosis-related genes and validated it in two independent melanoma cohorts. In addition, a nomogram combining the PGS score and clinicopathological characteristics of these cases was also established to accurately evaluate prognosis of melanoma^35^.

Melanoma shows a high rate of mutation with strong immunogenicity, which results in an abundant infiltration of the tumor microenvironment by immune cells^36^. The remodeling of an immune microenvironment can significantly affect the response of a tumor to systemic therapy and thus determine the prognosis of the patient^37–39^. We found in our present analyses that pyroptosis in melanoma was significantly positively correlated with the immune score, complement activation, IFN-γ response, TNF signaling and inflammatory response, strongly suggesting that occurrence of pyroptosis may induce the activation of immune responses in the melanoma microenvironment. Furthermore, we observed an elevated infiltration of various immune cells, including DC, M1 macrophages, CD4^+^ T cells, CD8^+^ T cells, NK cells, B cells, Th2 cells, and mast cells in our PGS-low score groups among the melanoma cases we analyzed.

Dendritic cells are specialized antigen-presenting cells that can effectively activate T cell immunity against melanoma^40–42^, and are significantly associated with an improved survival time in melanoma^43^. As the key trigger of pyroptosis, the NLRP3 inflammasome activated by dying tumor cells can stimulate DCs, thus effectively priming CD8^+^ T cells by secreting IL-1β, and thereby inducing a strong and durable tumor immune response^44^. Another study has found however that activation of the inflammasome with the pyroptosis agonist alum induced a weak DC function^45^. These findings indicate that the effect of pyroptosis on the functional activation of DCs in the melanoma microenvironment needs further exploration.

Apart from DCs, macrophages can phagocytose tumor cells and affect T cell function while performing an antigen presentation role. In the melanoma microenvironment, macrophages mainly manifest an M2-like pro-tumoral phenotype. Reprogramming M2 into M1 macrophages is also a potential therapeutic target for melanoma^46,47^. The expression of the pyroptosis executor GSDME can enhance the phagocytosis of macrophages and enable them to obtain an M1-like tumoricidal phenotype^48^. These results suggest that pyroptosis may also become a potential target for the reprogramming of tumor-associated macrophages.

It is well known that CD8^+^ cytotoxic T cells and NK cells can kill tumor cells directly. The infiltration and exerted functions of CD8^+^ T cells and NK cells are closely related to the prognosis and drug response in melanoma patients^49–51^. The pyroptosis executor GSDME can enhance the number and function of tumor-infiltrating CD8^+^T cells and NK cells^48^. The DAMPs, IL-1β and IL-18 released by pyroptosis of tumor cells can also effectively initiate CD8^+^ T cell expansion and promote NK cell recruitment, activate a tumor immune response, and improve the efficacy of immunotherapy^52–54^. Combined with our current findings, the evidence to date suggests therefore that the activation of a tumor-specific immune response by inducing pyroptosis may be an effective way to enhance the sensitivity to immunotherapy. With regard to CD4^+^T cells, B-cells and mast cells, their specific roles in the melanoma immune microenvironment remain unclear^35,55–57^. However, our present analyses have preliminarily uncovered a potential relationship between pyroptosis and these immune cell types, and thus provided a basis for further exploration of the mechanisms underlying this programmed cell death pathway that can affect their function.

In addition to the infiltration of the melanoma microenvironment by different immune cells, the expression of immune-related molecules can further reflect the response of melanoma to immunotherapy. In our current analyses, we found that pyroptosis is significantly associated with the increased expression of multiple immune checkpoint factors in the melanoma microenvironment, such as PD-1/PD-L1, CTLA4, TIM3, LAG3, and immune activation-related molecules, including OX-40, CD40, and CD86. The operation of immune checkpoints has been confirmed to affect the efficacy of immunotherapy through CD8^+^T cells^58^. PD-L1 or PD-1 expression in the tumor microenvironment is a logical biomarker for the prediction of the treatment response to anti-PD-1 or anti-PD-L1 therapies^59,60^. Moreover, various immune active molecules, such as CD86, CD40 and OX-40 can also potentially be used as biomarkers of immunotherapy efficacy in melanoma^58,61^. The close relationship between pyroptosis and the immune status of melanoma also suggests its potential as a biomarker of immunotherapeutic outcomes.

Given that the immune status of melanoma is associated with the therapeutic response of melanoma, our further analysis has revealed that pyroptosis is associated with enhanced sensitivity to multiple agents, such as paclitaxel, cisplatin, imatinib and interferon-α. The *SLC31A2* and *COL4A5* genes were identified to be associated with tumor resistance to most chemotherapies. In vitro experiments have also demonstrated that the resistance of B16F10 melanoma cells to vemurafenib was tightly related to these two genes. Previous studies have additionally found that inducing pyroptosis can overcome the resistance of melanoma cells to targeted therapies^62,63^.Also, pyroptosis can enhance the sensitivity of melanoma cells to doxorubicin^64^. These prior findings provide insights into the future use of pyroptosis as a biomarker of the efficacy of combination therapy strategies. However, our current study was limited by the incompleteness of the drug database and the fact that we did not analyze the effect of pyroptosis on the sensitivity of commonly used targeted therapeutic and immunotherapeutic agents in melanoma patients.

In summary, our work identified undiscovered pyroptosis-related genes and an original PGS, which could be a more accurate and novelty tool in not only predicting melanoma prognosis, but also guiding personalized treatment.

## Materials and methods

### Dataset acquisition

The expression profiles of melanoma patients in the GSE54467 and GSE19234 datasets were downloaded from the GEO dataset (http://www.ncbi.nlm.nih.gov/geo). The GSE54467 dataset contained 77 melanoma patients and GSE19234 dataset included 44 melanoma samples. The expression data and clinical information for 455 melanoma samples in the TCGA cohort were downloaded from the UCSC Xena website (http://xena.ucsc.edu). The GSE54467 dataset was utilized as the training cohort for establishing the prognosis model, which was then validated in the other two cohorts, including GSE19234 and TCGA cohort. The three cohorts were are independent of each other. Raw data were standardized and normalized using the R software version 4.0.3 (https://www.r-project.org/).

### Single sample gene set enrichment analysis

Single sample gene set enrichment analysis (ssGSEA) was performed using R package “GSVA”. The enrichment in tumor-related pathways in the GSE54467 database was explored with this method. Tumor-related pathways and drug response gene sets were obtained from the MSigDB database (http://www.gsea-msigdb.org).

### Weighted gene co-expression network analysis

We extracted the 25% of genes with the largest variance from the gene expression datasets in GSE54467 to perform WGCNA. The R package component “WGCNA” was used to identify pyroptosis trait-related modules. The soft-thresholding power was set at 7 to transform the similarity gene matrix expression into an adjacency matrix. 0.80 was set as the fitting degree of the scale-free topological model. To optimize the dependability of the results, 30 was set as the minimum number of genes. Genes with a p value of less than 0.01 were extracted for further analysis as pyroptosis-related genes and modules.

### Gene set enrichment analysis

Gene set enrichment analysis (GSEA) was used for functional assessments of the identified pyroptosis-related genes. Chip expression profiles and sample data files were separately created from the training cohort and all validation cohorts and then imported into the GSEA software 4.2.1 (https://www.gsea-msigdb.org/gsea/index.jsp). A false discovery rate (FDR) < 0.25 and p < 0.05 were defined as significant.

### Construction and validation of melanoma prognostic predictive signature

Univariate and multivariate Cox regression analysis were used to identify overall survival (OS)-related cancer hallmarks. Lasso Cox regression analysis was employed to select pyroptosis-related genes. Finally, to identify genes that are tightly associated with pyroptosis and establish the prognostic PGS, the LASSO Cox regression model was utilized. For each sample, the coefficients of Logistic Regression were applied to calculate the PGS score as follows: PGS score = ∑ (coefficient × mRNA expression).

### Establishment of a nomogram for melanoma prognosis prediction

The PGS score and correlative clinical parameters were used to establish a nomogram via the “rms” and “survival” packages within the R software. This nomogram was established to evaluate the 1-, 3-, and 5-year survival probabilities. The performance of the model was assessed by calibration curve and C index.

### Immune and stromal cell infiltration analysis

To assess the comprehensive tumor environment for melanoma, xCell was used to calculate the immune^65^, stromal cell scores and infiltration levels of 48 tumor microenvironment-related cell categories of each patient in the GSE54467 dataset. The infiltration levels of 22 immune cell types in each patient were evaluated using the CIBERSORTx online website (https://cibersortx.stanford.edu)^66^. The R package “MCPcounter” was used to calculate the absolute abundance of 10 immune cell populations infiltrating the tissue^67^. Specific cell types selected in different methods were exhibited in Supplementary Table S7.

### Drug sensitivity analysis

The Genomics of Drug Sensitivity in Cancer database was employed to conduct drug sensitivity analysis (https://www.cancerrxgene.org). The interrelation between the expression of 12 target genes and drug sensitivity was determined by Spearman correlation analysis.

### Cell culture and transfection

B16F10 melanoma cell line was cultured in Dulbecco’s modified Eagle’s medium (DMEM) containing 1% antibiotic–antimycotic solution and supplemented with Zelanian-certified 10% fetal bovine serum. The cells were placed in a humidified incubator in a 5% CO_2_ atmosphere at 37 °C. The DNA sequences encoding the small interfering RNAs (siRNAs) used to target *SLC31A2* and *COL4A5* were as follows: siSLC31A2 (forward, 5′-CCA GAU CAA CUU CAG ACA ATT-3′ and reverse, 5′-UUG UCU GAA GUU GAU CUG GTT-3′) and siCOL4A5 (forward, 5′-GAC AGA GUA UUG UAA UCA ATT-3′ and reverse, 5′-UUG AUU ACA AUA CUC UGU CTT-3′). Transfection of the siSLC31A2 and siCOL4A5 constructs was conducted using Lipofectamine 2000 reagent (ThermoFisher, Shanghai, China) following the manufacturer’s instructions.

### Cell proliferation assay

B16F10 melanoma cell line was transfected with control siRNA (siCon), siSLC31A2, or siCOL4A5, or untreated, and cultured separately in 96-well plates. At 24 h later, the cells were treated with vemurafenib (Aladdin Reagent, Shanghai, China) at 2.5–2500.0 ng/mL for a further 24 h. The CCK-8 assay was used for the cell proliferation assay following the manufacturer's instructions. The optical densities of the cell solutions were assessed at 450 nm (OD450) using a Multiskan™ FC plate reader (ThermoFisher Scientific, Shanghai, China). The mean results of three independent wells were calculated.

### Statistical analysis

Data were statistically analyzed using R software. The hazard ratio (HR) of forest plots was measured by univariate Cox or multivariate Cox proportional hazard regression. Survival analysis was conducted with Kaplan–Meier curves. A Wilcoxon test was applied to assess differences between two groups. Values of *p* < 0.05 were considered significant.

## Supplementary Information

Supplementary Tables.

Supplementary Figures.

## Data Availability

The datasets used and analyzed during the current study are available from the corresponding author on reasonable request.
