# Supplementary material for: The expression pattern of pyroptosis-related genes predicts the prognosis and drug response of melanoma
Source: Sci Rep. 2022 Dec 13;12:21566. doi: 10.1038/s41598-022-24879-y (PMC9747972; doi:10.1038/s41598-022-24879-y)
Supplement: Supplementary file 2 — Supplementary Figures. [file 41598_2022_24879_MOESM2_ESM.docx]

**Supplementary Figures**


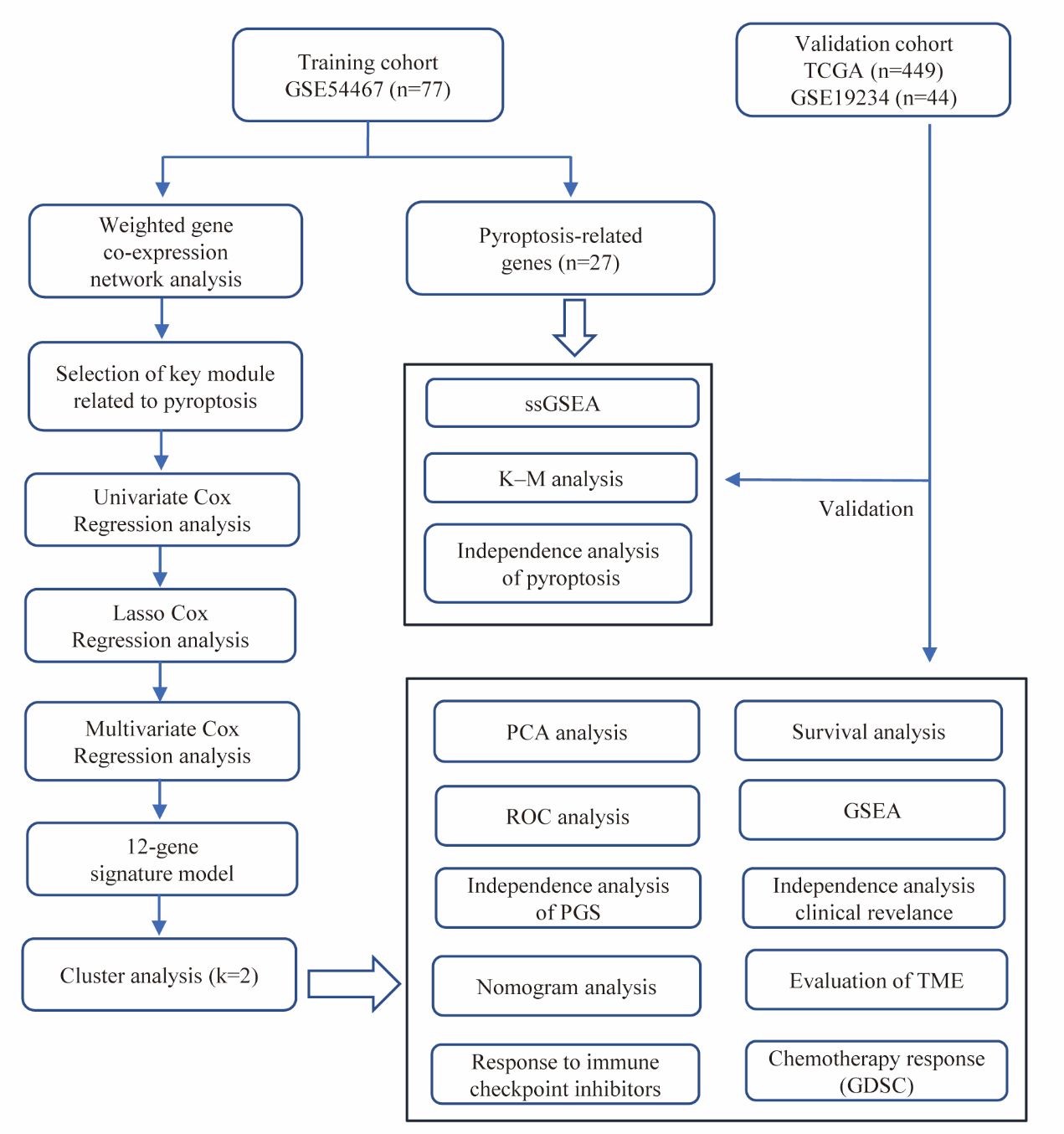


**Supplementary Figure S1.** Study flowchart.


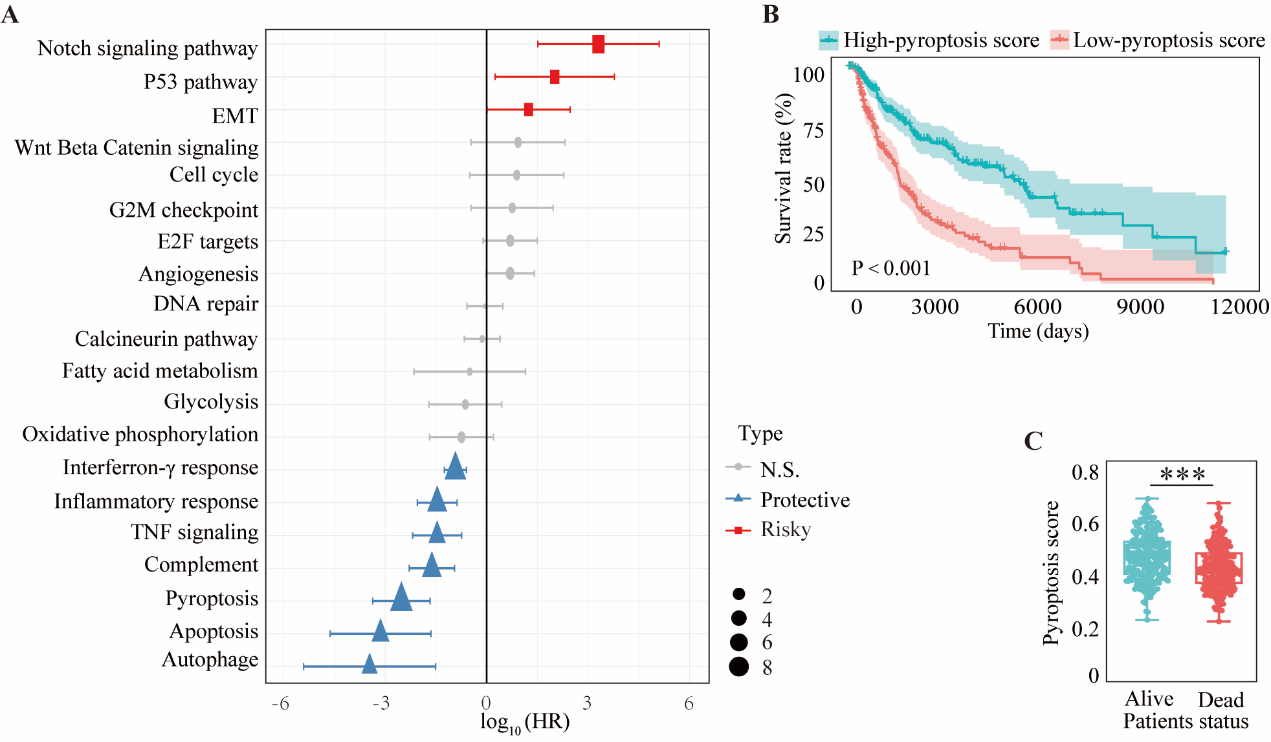


**Supplementary Figure S2.** Confirmation of the association of pyroptosis with the overall survival (OS) outcomes among the melanoma patients in the TCGA dataset. (A) Hazard ratios (HR) for 20 hallmarks of cancer prognosis determined using a forest plot. (B) Kaplan–Meier curves for the patients with high and low pyroptosis scores. (C) Comparison of the pyroptosis scores in the living and deceased patients. Statistical analysis: *** *p* < 0.001. This figure was created using R software version 4.0.3 (<https://www.r-project.org/>).


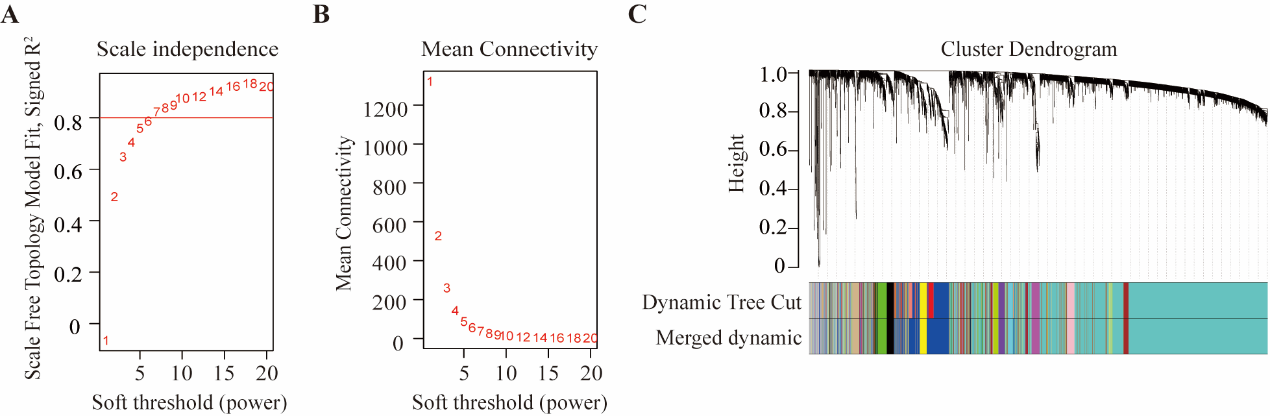


**Supplementary Figure S3.** Selection of the optimal soft-threshold power and clustered dendrogram of the genes identified by WGCNA. (A) Calculation of the scale-free index for different soft-threshold powers (β). (B) Calculation of the mean connectivity for different soft-threshold powers. (C) Clustered dendrogram for differentially expressed genes. This figure was created using R software version 4.0.3 (<https://www.r-project.org/>).


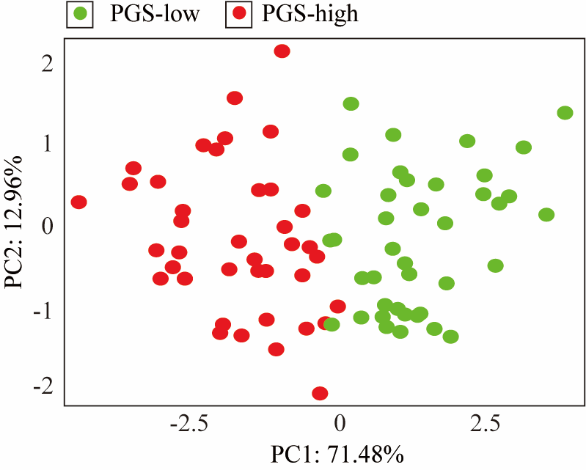


**Supplementary Figure S4.** Principal component analysis for the expression of pyroptosis-related regulators that distinguish the PGS-low (n = 39) from the PGS-high (n = 38) melanoma patient groups in the GSE54467 database. This figure was created using R software version 4.0.3 (<https://www.r-project.org/>).


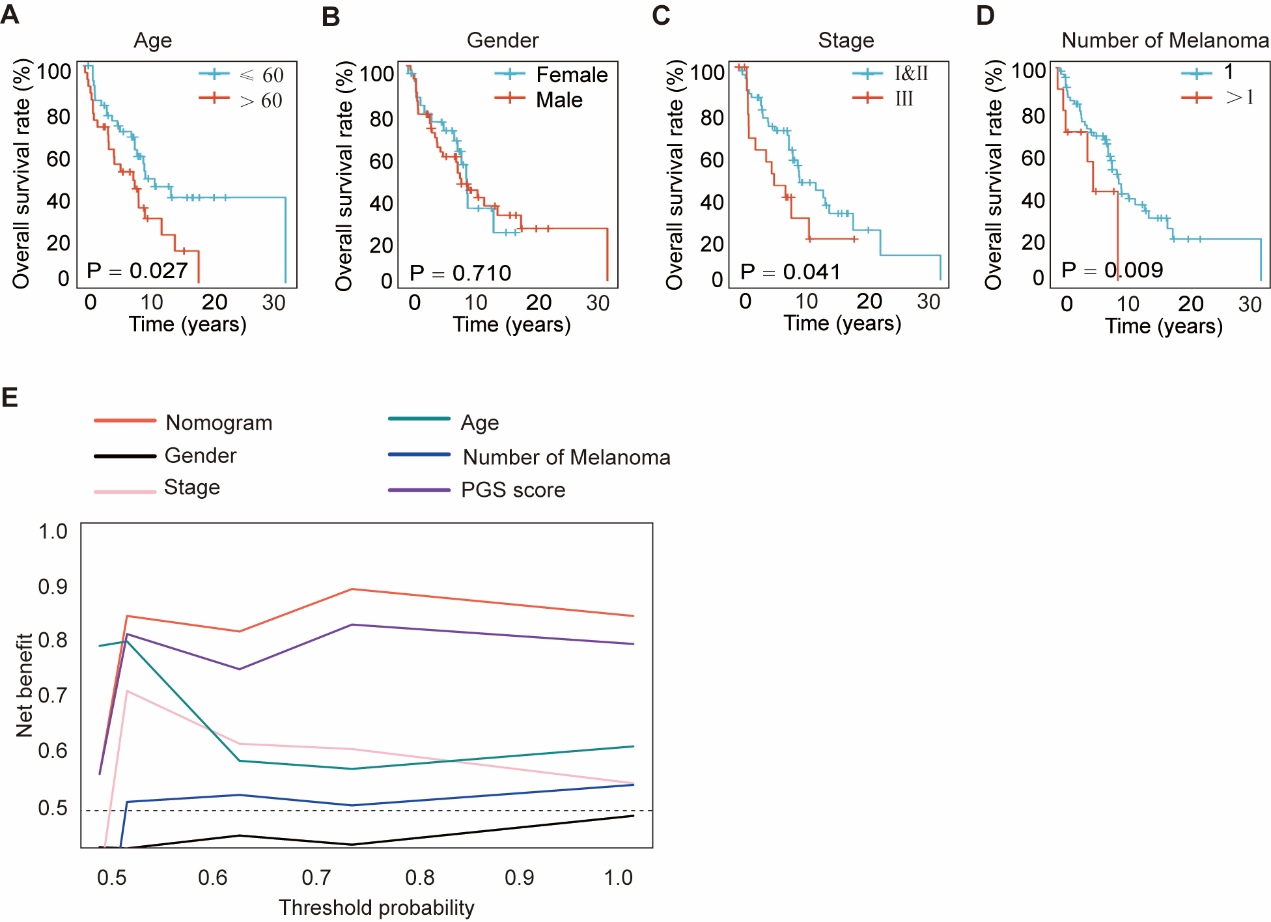


**Supplementary Figure S5.** Kaplan-Meier OS curves for (A) Age, (B) Gender, (C) Stage and (D) number of melanomas. (E) Decision curve analysis shows roles of clinicopathological parameters predicting OS on the basis of the nomogram. This figure was created using R software version 4.0.3 (<https://www.r-project.org/>).


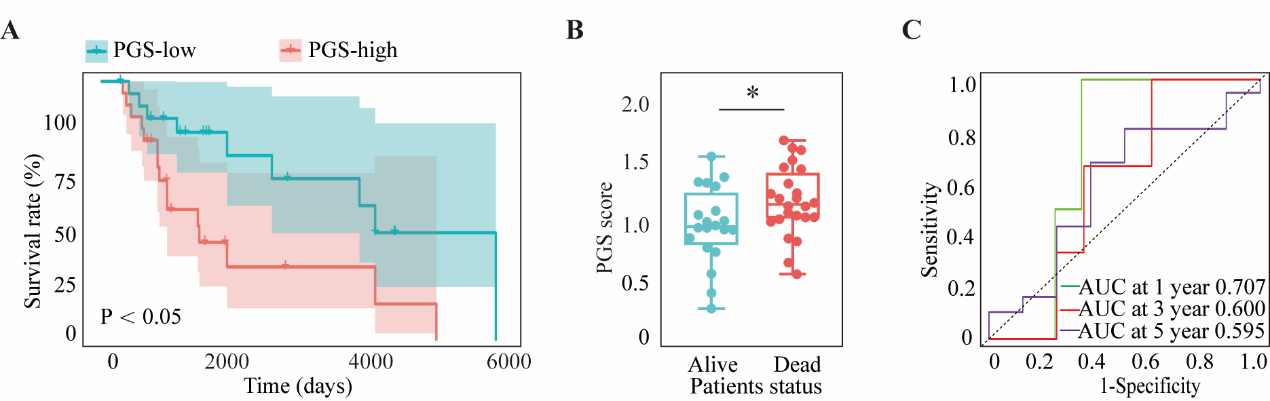


**Supplementary Figure S6.** Validation of the PGS score in the GSE19234 dataset. (A) Kaplan–Meier OS curves for the melanoma patients in PGS-high and -low groups. (B) Comparison of the PGS scores in the living and deceased patients. (C) Time-dependent ROC curves at 1, 3 and 5 years. Statistical analysis: * *p* < 0.05. This figure was created using R software version 4.0.3 (<https://www.r-project.org/>).


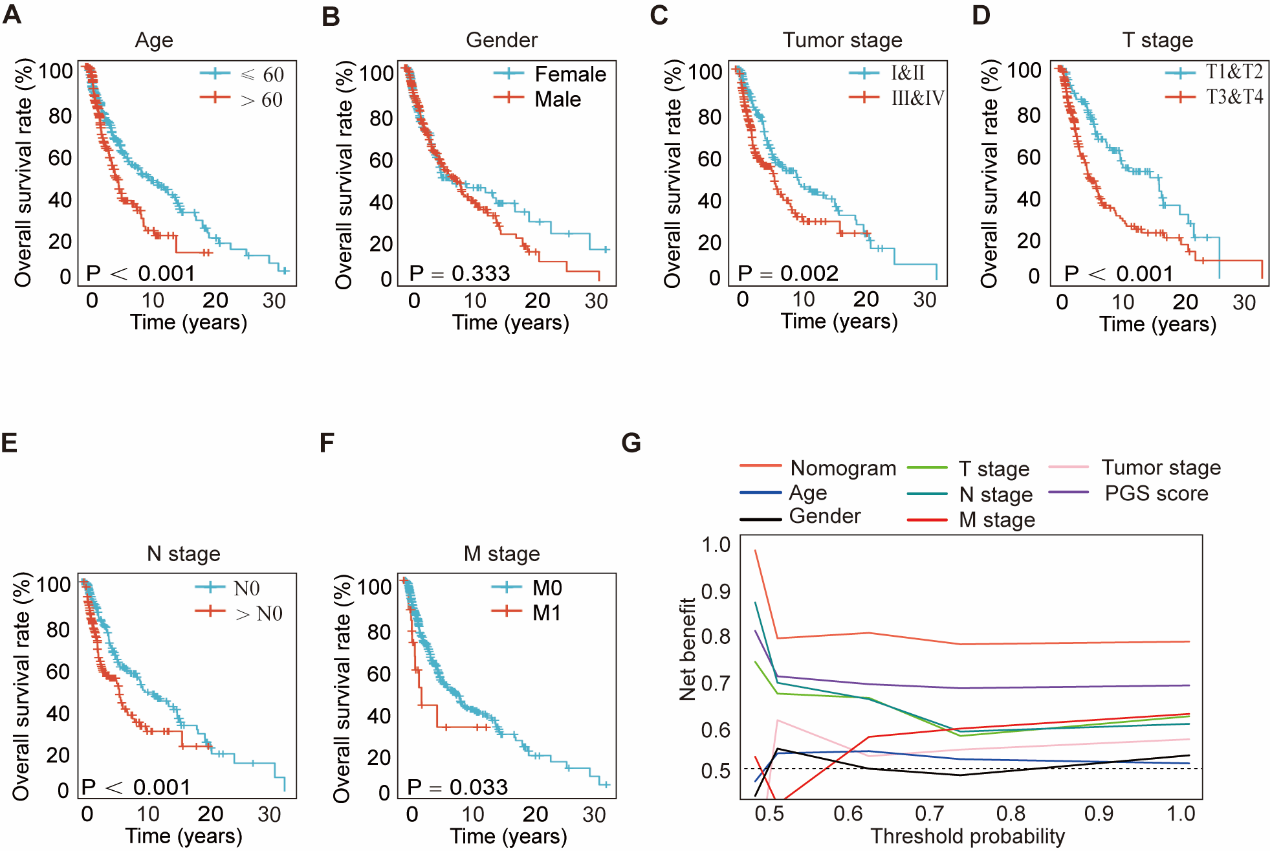


**Supplementary Figure S7.** Kaplan-Meier OS curves for (A) Age, (B) Gender, (C) Tumor stage, (D) T stage, (E) T stage, and (F) T stage. (G) Decision curve analysis shows roles of clinicopathological parameters predicting OS on the basis of the nomogram. This figure was created using R software version 4.0.3 (<https://www.r-project.org/>).


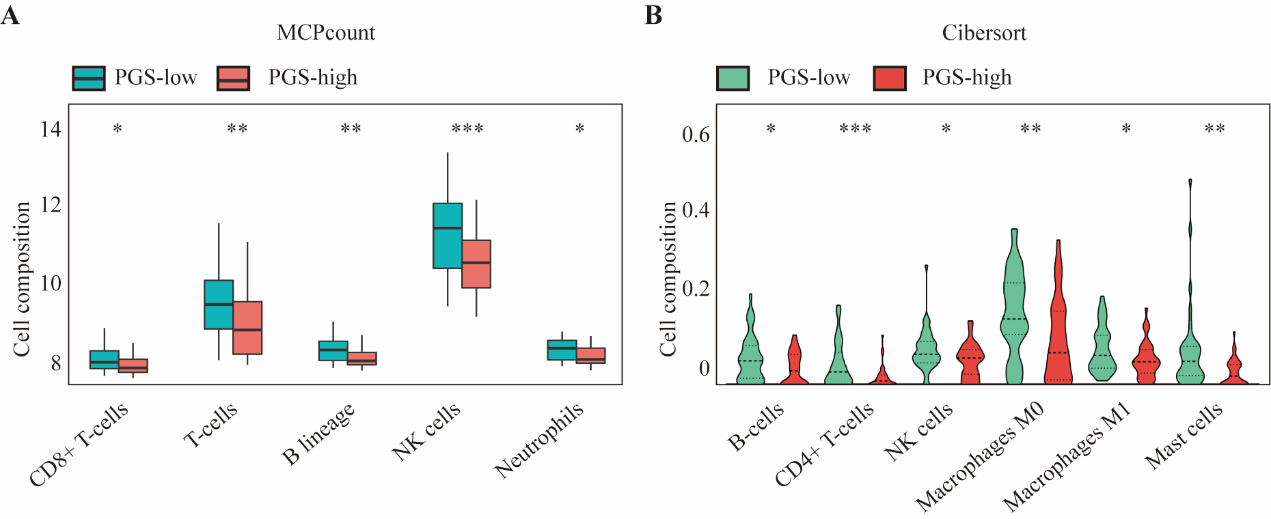


**Supplementary Figure S8.** (A-B) Evaluation of immune cell infiltration using MCPcounter and Cibersort in the PGS-low and -high groups. Statistical analysis: * *p* < 0.05, ** *p* < 0.01, *** *p* < 0.001. This figure was created using R software version 4.0.3 (<https://www.r-project.org/>).


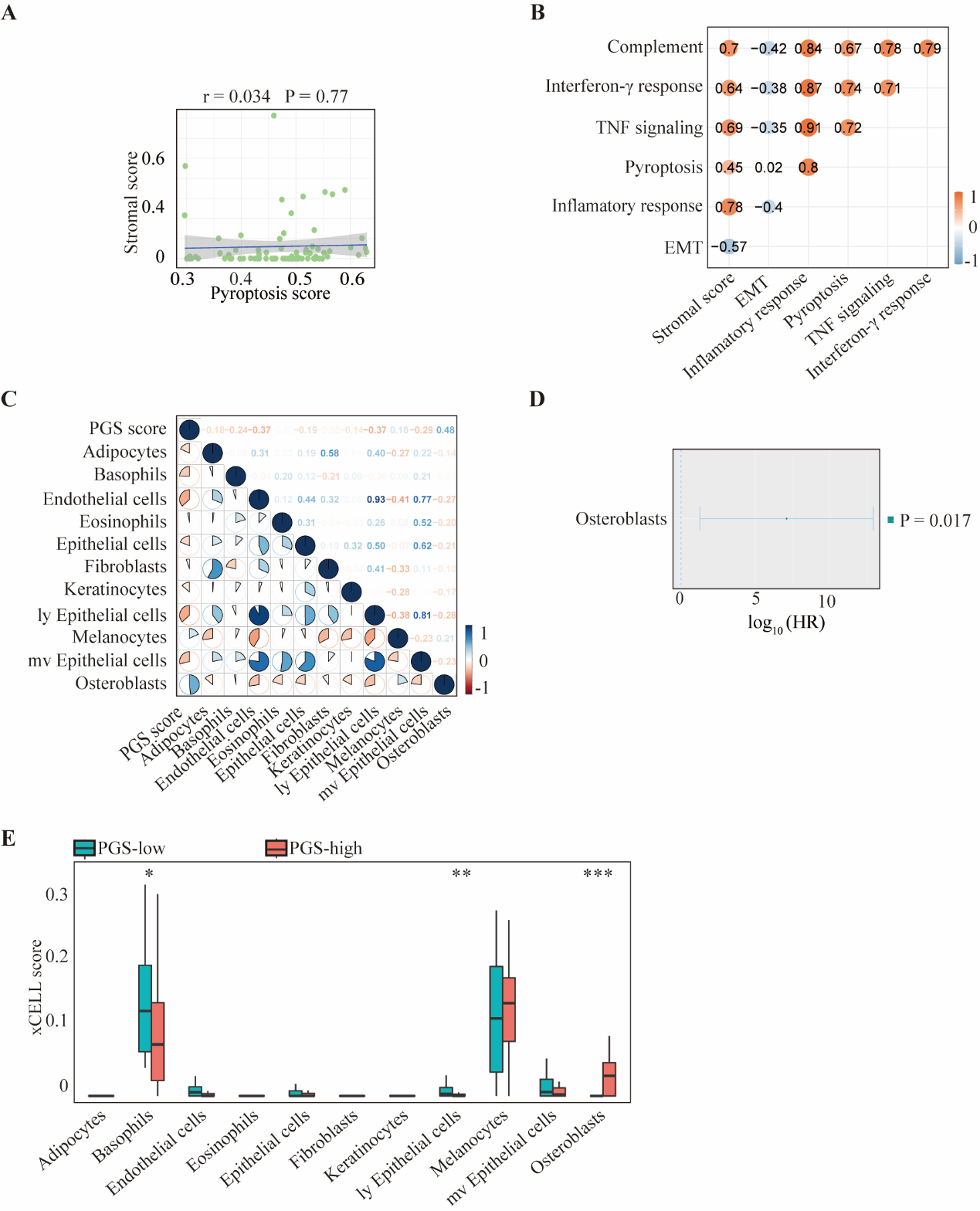


**Supplementary Figure S9.** Correlation between the PGS score and stromal cell infiltration. (A) The interrelationship between stromal score and pyroptosis score. (B)  Interrelationship between pyroptosis and cancer hallmarks. (C) Interrelationship between the PGS score and various stromal cell types. (D) Multivariate Cox regression analysis to identify various prognostic stromal cell types in a HR forest plot. (E)  Infiltration level of 11 stromal cell types in the PGS-low and -high groups. Statistical analysis: * *p* < 0.05, ** *p* < 0.01, *** *p* < 0.001. This figure was created using R software version 4.0.3 (<https://www.r-project.org/>).
